# 2,4-Dibromo-6-[(quinolin-8-yl­amino)­methyl­idene]cyclo­hexa-2,4-dien-1-one monohydrate

**DOI:** 10.1107/S1600536811048793

**Published:** 2011-11-19

**Authors:** Keisuke Kawamoto, Takashi Shibahara

**Affiliations:** a Department of Chemistry, Okayama University of Science, Ridai-cho, Okayama 700-0005, Japan

## Abstract

In the title compound, C_16_H_10_Br_2_N_2_O·H_2_O, bifurcated intra­molecular N—H⋯(N,O) hydrogen bonding defines the essential planarity of the main mol­ecule: the dihedral angle between the quinoline and benzene rings is 7.53 (8)°. Inter­molecular O—H⋯O and weak C—H⋯O hydrogen bonds consolidate the crystal packing, which exhibits π–π inter­actions with a distance of 3.588 (1) Å between the centroids of the aromatic rings and short Br⋯Br contacts of 3.5757 (6) Å.

## Related literature

For a related structure, see: Shibahara *et al.* (2010 ▶).
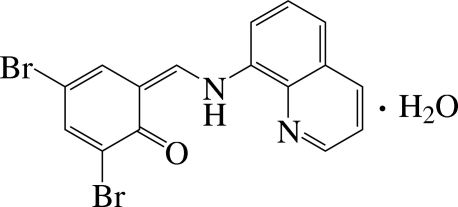


## Experimental

### 

#### Crystal data

C_16_H_10_Br_2_N_2_O·H_2_O
*M* *_r_* = 424.09
Triclinic, 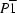

*a* = 7.0027 (8) Å
*b* = 8.2782 (11) Å
*c* = 12.9164 (19) Å
α = 94.953 (9)°
β = 102.010 (5)°
γ = 94.551 (6)°
*V* = 725.99 (17) Å^3^
*Z* = 2
Mo *K*α radiation
μ = 5.61 mm^−1^
*T* = 93 K
0.64 × 0.58 × 0.18 mm

#### Data collection

Rigaku Mercury70 diffractometer
Absorption correction: multi-scan (*REQAB*; Rigaku, 1998 ▶) *T*_min_ = 0.145, *T*_max_ = 0.364
6768 measured reflections
3281 independent reflections
3070 reflections with *F* ^2^ > 2σ(*F* ^2^)
*R*_int_ = 0.049

#### Refinement

*R*[*F* ^2^ > 2σ(*F* ^2^)] = 0.038
*wR*(*F* ^2^) = 0.104
*S* = 1.00
3281 reflections
239 parameters
3 restraints
H-atom parameters constrained
Δρ_max_ = 1.13 e Å^−3^
Δρ_min_ = −1.49 e Å^−3^

### 

Data collection: *CrystalClear* (Rigaku, 2007 ▶); cell refinement: *CrystalClear*; data reduction: *CrystalClear*; program(s) used to solve structure: *SHELXS97* (Sheldrick, 2008 ▶); program(s) used to refine structure: *SHELXL97* (Sheldrick, 2008 ▶); molecular graphics: *CrystalStructure* (Rigaku, 2010 ▶); software used to prepare material for publication: *CrystalStructure*.

## Supplementary Material

Crystal structure: contains datablock(s) global, I. DOI: 10.1107/S1600536811048793/cv5187sup1.cif

Structure factors: contains datablock(s) I. DOI: 10.1107/S1600536811048793/cv5187Isup2.hkl

Supplementary material file. DOI: 10.1107/S1600536811048793/cv5187Isup3.cml

Additional supplementary materials:  crystallographic information; 3D view; checkCIF report

## Figures and Tables

**Table 1:** Hydrogen-bond geometry (Å, °)

*D*—H⋯*A*	*D*—H	H⋯*A*	*D*⋯*A*	*D*—H⋯*A*
O2—H2*a*⋯O1	0.95	1.86	2.802 (4)	171
N1—H4⋯O1	0.83	1.90	2.592 (4)	140
N1—H4⋯N2	0.83	2.31	2.668 (3)	107
C7—H3⋯O2^i^	0.95	2.31	3.234 (4)	166

Symmetry code: (i) 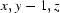.

**Fig. 1.**
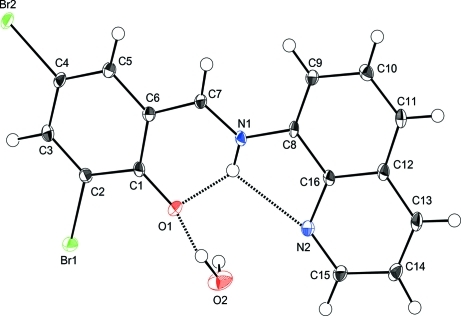
The molecular structure of (I).H2O with the atom–labeling scheme. Displacement ellipsoids are drawn at the 50% probability level. Hydrogen bonds are shown as dashed lines.

## supplementary crystallographic information

### Comment

The title dibromo compound (I) was obtained by the condensation reaction of salicylaldehyde with 8-aminoquinoline. We have reported the synthesis and X-ray structure of the corresponding dichloro compound 2,4-Dichloro-6-(8-quinolylaminomethylene)cyclohexa-2,4-dien-1-one methanol solvate (II).methanol (Shibahara *et al.*, 2010). The compounds (I) and (II) exist in the keto form and the C=O and N—H bonds are mutually *cis* in the crystal structure (Fig. 1). The dihedral angle between the quinoline and benzene rings in (I) [7.53 (8)°] is smaller than that in (II) [11.17 (3)°]. The crystal packing of (I) exhibits π-π interactions with the distance of 3.588 (1) Å between the centroids of aromatic rings.

In the crystal structure of (I).H_2_O, H_2_O molecule are linked through intermolecular O—H···O, weak C—H···O hydrogen bonds (Table 1). There are short Br···Br contacts of 3.5757 (6) Å (the sum of van der Waals radii of Br, 3.70 Å). In addition, the O···O distance (2.981 (4) Å) between two adjacent water molecules is shorter than the sum (3.04 Å) of van der Waals radii.

### Experimental

Refluxing a suspension of 8-aminoquinoline (104 mg, 0.7 mmol) and 3,5-dibromo-salicylaldehyde (200 mg, 0.7 mmol) in acetonitrile (2 ml) at 65°C for three hours gave red powder, C_16_H_10_Br_2_N_2_O (I), Yield 256 mg (83%). Anal. Found: C, 47.27; H, 2.05; N, 6.69%. Calcd for C_16_H_10_Br_2_N_2_O (I): C, 47.32; H, 2.48; N, 6.90%. Orange block single crystals of (I).H_2_O suitable for X-ray analysis were obtained by dissolving 10 mg of (I) in acetonitrile (10 ml) containing small amount of water (*ca* 0.2%) followed by the slow evaporation of the acetonitrile solution Yield 90%. Anal. Found: C, 45.51; H, 2.76; N, 6.34%. Calcd for C_16_H_10_Br_2_N_2_O (I).H_2_O: C, 45.31; H, 2.85; N, 6.61%.

### Refinement

H atoms were geometrically positioned (O—H 0.95 Å; C—H 0.94-0.98 Å; N—H 0.83 Å), and refined as riding, with U_iso_(H) = 1.2 U_eq_ of the parent atom.

### Crystal data

**Table:**

C_16_H_10_Br_2_N_2_O·H_2_O	*Z* = 2
*M**_r_* = 424.09	*F*(000) = 416.00
Triclinic, *P*1	*D*_x_ = 1.940 Mg m^−^^3^
Hall symbol: -P 1	Mo *K*α radiation, λ = 0.71075 Å
*a* = 7.0027 (8) Å	Cell parameters from 2134 reflections
*b* = 8.2782 (11) Å	θ = 3.1–30.0°
*c* = 12.9164 (19) Å	µ = 5.61 mm^−^^1^
α = 94.953 (9)°	*T* = 93 K
β = 102.010 (5)°	Block, orange
γ = 94.551 (6)°	0.64 × 0.58 × 0.18 mm
*V* = 725.99 (17) Å^3^	

### Data collection

**Table:**

Rigaku Mercury70 diffractometer	3070 reflections with *F*^2^ > 2σ(*F*^2^)
Detector resolution: 7.314 pixels mm^-1^	*R*_int_ = 0.049
ω scans	θ_max_ = 27.5°
Absorption correction: multi-scan (*REQAB*; Rigaku, 1998)	*h* = −9→9
*T*_min_ = 0.145, *T*_max_ = 0.364	*k* = −10→10
6768 measured reflections	*l* = −16→16
3281 independent reflections	

### Refinement

**Table:**

Refinement on *F*^2^	Secondary atom site location: difference Fourier map
*R*[*F*^2^ > 2σ(*F*^2^)] = 0.038	Hydrogen site location: inferred from neighbouring sites
*wR*(*F*^2^) = 0.104	H-atom parameters constrained
*S* = 1.00	*w* = 1/[σ^2^(*F*_o_^2^) + (0.0485*P*)^2^ + 1.155*P*] where *P* = (*F*_o_^2^ + 2*F*_c_^2^)/3
3281 reflections	(Δ/σ)_max_ < 0.001
239 parameters	Δρ_max_ = 1.13 e Å^−^^3^
3 restraints	Δρ_min_ = −1.49 e Å^−^^3^
Primary atom site location: structure-invariant direct methods	

### Special details

**Table:**

Geometry. The dihedral angle between the quinoline rings (C8~C16, N2) and the benzene rings (C1~C6) is 7.53 (8)*^o^*: Mean derivations of the atoms from the former and latter planes are 0.004 and 0.015 Å, respectively.
Refinement. Refinement was performed using all reflections. The weighted *R*-factor (*wR*) and goodness of fit (*S*) are based on *F*^2^. *R*-factor (gt) are based on *F*. The threshold expression of *F*^2^ > 2.0 σ(*F*^2^) is used only for calculating *R*-factor (gt).

### Fractional atomic coordinates and isotropic or equivalent isotropic displacement parameters (Å^2^)

**Table:**

	*x*	*y*	*z*	*U*_iso_*/*U*_eq_	
Br2	−0.34236 (4)	−0.27358 (3)	0.49272 (2)	0.01502 (11)	
Br1	−0.11289 (4)	0.39307 (3)	0.60673 (2)	0.01673 (11)	
O2	0.0121 (4)	0.5645 (3)	0.8971 (3)	0.0284 (6)	
O1	0.1087 (3)	0.2673 (3)	0.81099 (16)	0.0151 (4)	
N1	0.2083 (4)	0.0514 (3)	0.94081 (18)	0.0110 (5)	
N2	0.4089 (4)	0.3076 (3)	1.06739 (19)	0.0134 (5)	
C9	0.2975 (4)	−0.1272 (4)	1.0815 (3)	0.0125 (5)	
C10	0.3944 (4)	−0.1428 (4)	1.1866 (3)	0.0139 (6)	
C11	0.4926 (4)	−0.0104 (4)	1.2520 (2)	0.0138 (5)	
C12	0.5002 (4)	0.1450 (4)	1.2159 (2)	0.0123 (5)	
C16	0.4041 (4)	0.1628 (4)	1.1104 (2)	0.0109 (5)	
C13	0.6009 (4)	0.2872 (4)	1.2787 (3)	0.0155 (6)	
C14	0.6063 (4)	0.4315 (4)	1.2356 (3)	0.0165 (6)	
C15	0.5084 (4)	0.4354 (4)	1.1284 (3)	0.0145 (6)	
C8	0.3016 (4)	0.0233 (4)	1.0440 (2)	0.0103 (5)	
C7	0.1134 (4)	−0.0578 (4)	0.8644 (2)	0.0112 (5)	
C6	0.0197 (4)	−0.0169 (4)	0.7638 (2)	0.0112 (5)	
C5	−0.0839 (4)	−0.1449 (4)	0.6883 (3)	0.0123 (5)	
C4	−0.1845 (4)	−0.1088 (4)	0.5924 (2)	0.0121 (5)	
C3	−0.1870 (4)	0.0531 (4)	0.5671 (3)	0.0133 (5)	
C2	−0.0905 (4)	0.1771 (4)	0.6392 (2)	0.0121 (5)	
C1	0.0202 (4)	0.1516 (4)	0.7428 (2)	0.0112 (5)	
H1	−0.2620	0.0797	0.4991	0.0342*	
H2	−0.0912	−0.2521	0.7072	0.0161*	
H4	0.2139	0.1456	0.9242	0.0275*	
H5	0.2367	−0.2221	1.0381	0.0106*	
H6	0.3890	−0.2477	1.2091	0.0202*	
H7	0.5539	−0.0174	1.3234	0.0211*	
H8	0.6675	0.2841	1.3519	0.0097*	
H9	0.6727	0.5322	1.2716	0.0153*	
H2a	0.0342	0.4649	0.8612	0.0548*	
H2b	−0.1246	0.5552	0.8955	0.1042*	
H10	0.5148	0.5362	1.0986	0.0174*	
H3	0.1074	−0.1692	0.8772	0.0135*	

### Atomic displacement parameters (Å^2^)

**Table:**

	*U*^11^	*U*^22^	*U*^33^	*U*^12^	*U*^13^	*U*^23^
Br2	0.02026 (17)	0.01242 (17)	0.00997 (16)	0.00054 (11)	0.00004 (11)	−0.00334 (11)
Br1	0.02187 (18)	0.01128 (17)	0.01506 (17)	−0.00026 (11)	−0.00019 (12)	0.00230 (12)
O2	0.0317 (13)	0.0174 (12)	0.0381 (15)	0.0017 (9)	0.0168 (11)	−0.0071 (11)
O1	0.0196 (10)	0.0114 (9)	0.0125 (10)	−0.0009 (8)	0.0020 (8)	−0.0027 (8)
N1	0.0143 (10)	0.0124 (11)	0.0077 (11)	0.0023 (9)	0.0054 (8)	0.0003 (9)
N2	0.0162 (11)	0.0138 (11)	0.0106 (11)	0.0008 (9)	0.0049 (9)	−0.0005 (9)
C9	0.0154 (12)	0.0125 (13)	0.0103 (12)	0.0012 (10)	0.0056 (10)	−0.0013 (10)
C10	0.0170 (13)	0.0147 (13)	0.0124 (13)	0.0037 (10)	0.0080 (10)	0.0014 (11)
C11	0.0145 (12)	0.0200 (14)	0.0073 (12)	0.0034 (10)	0.0031 (10)	−0.0000 (11)
C12	0.0119 (12)	0.0176 (14)	0.0086 (12)	0.0018 (10)	0.0055 (9)	−0.0011 (10)
C16	0.0126 (12)	0.0138 (13)	0.0082 (12)	0.0024 (9)	0.0069 (9)	−0.0000 (10)
C13	0.0158 (12)	0.0220 (15)	0.0084 (12)	0.0008 (11)	0.0040 (10)	−0.0020 (11)
C14	0.0170 (13)	0.0196 (15)	0.0128 (13)	−0.0022 (11)	0.0070 (10)	−0.0050 (11)
C15	0.0179 (13)	0.0135 (13)	0.0126 (13)	0.0003 (10)	0.0067 (10)	−0.0029 (11)
C8	0.0114 (11)	0.0143 (13)	0.0060 (12)	0.0033 (10)	0.0046 (9)	−0.0021 (10)
C7	0.0133 (11)	0.0117 (12)	0.0093 (12)	0.0007 (10)	0.0048 (9)	−0.0012 (10)
C6	0.0129 (12)	0.0117 (13)	0.0102 (12)	0.0017 (9)	0.0058 (9)	−0.0003 (10)
C5	0.0145 (12)	0.0120 (13)	0.0112 (13)	0.0024 (10)	0.0050 (10)	−0.0010 (10)
C4	0.0138 (12)	0.0130 (13)	0.0093 (12)	−0.0001 (10)	0.0044 (9)	−0.0038 (10)
C3	0.0156 (12)	0.0160 (14)	0.0094 (12)	0.0024 (10)	0.0052 (10)	0.0007 (10)
C2	0.0147 (12)	0.0115 (12)	0.0105 (12)	0.0020 (10)	0.0030 (10)	0.0017 (10)
C1	0.0138 (12)	0.0129 (13)	0.0079 (12)	0.0016 (9)	0.0047 (9)	0.0001 (10)

### Geometric parameters (Å, °)

**Table:**

Br2—C4	1.899 (3)	C6—C1	1.444 (4)
Br1—C2	1.883 (3)	C5—C4	1.362 (4)
O1—C1	1.269 (3)	C4—C3	1.407 (4)
N1—C8	1.405 (4)	C3—C2	1.357 (4)
N1—C7	1.308 (4)	C2—C1	1.441 (4)
N2—C16	1.365 (4)	O2—H2a	0.950
N2—C15	1.318 (4)	O2—H2b	0.950
C9—C10	1.408 (4)	N1—H4	0.827
C9—C8	1.375 (4)	C9—H5	0.940
C10—C11	1.366 (4)	C10—H6	0.940
C11—C12	1.406 (5)	C11—H7	0.941
C12—C16	1.415 (4)	C13—H8	0.966
C12—C13	1.417 (4)	C14—H9	0.953
C16—C8	1.423 (4)	C15—H10	0.950
C13—C14	1.362 (5)	C7—H3	0.950
C14—C15	1.417 (4)	C5—H2	0.941
C7—C6	1.411 (4)	C3—H1	0.977
C6—C5	1.416 (4)		
			
Br1···O1	3.079 (2)	N1···H5^v^	3.5727
O1···N1	2.592 (4)	N2···H5^vi^	3.1676
O1···N2	3.507 (3)	N2···H2b^iii^	2.4795
O1···C7	2.836 (4)	N2···H10^iv^	2.7204
N1···N2	2.668 (3)	C9···H4^v^	3.5576
N1···C1	2.846 (4)	C9···H4^vi^	3.4525
N2···C13	2.806 (4)	C9···H10^i^	3.2816
C9···C12	2.810 (4)	C10···H4^vi^	3.3478
C9···C7	2.957 (4)	C10···H9^i^	3.5628
C10···C16	2.793 (5)	C10···H10^i^	3.0491
C11···C8	2.789 (4)	C11···H1^xi^	3.2864
C12···C15	2.746 (5)	C11···H4^vi^	3.5395
C16···C14	2.738 (4)	C12···H2^v^	3.3776
C15···C8	3.584 (4)	C12···H3^vi^	3.2196
C6···C3	2.782 (4)	C16···H5^vi^	3.4957
C5···C2	2.794 (4)	C16···H2b^iii^	3.1593
C4···C1	2.847 (4)	C16···H3^vi^	3.3870
Br2···Br1^i^	3.5758 (6)	C13···H1^xi^	3.4564
Br1···Br2^ii^	3.5758 (6)	C13···H2^vi^	3.4377
O2···O2^iii^	2.981 (4)	C13···H3^vi^	3.2974
O2···O1	2.802 (4)	C14···H6^ii^	3.1729
O2···N2^iii^	3.317 (4)	C14···H2a^iv^	3.1287
O2···C9^ii^	3.513 (4)	C14···H2b^iii^	3.4607
O2···C14^iv^	3.461 (5)	C14···H3^vi^	3.4954
O2···C15^iii^	3.591 (4)	C15···H5^vi^	3.5171
O2···C15^iv^	3.441 (4)	C15···H6^ii^	2.9843
O2···C7^ii^	3.235 (4)	C15···H2a^iv^	3.2156
O1···O2	2.802 (4)	C15···H2b^iii^	2.6491
O1···C9^v^	3.573 (4)	C15···H10^iv^	2.9333
O1···C10^v^	3.599 (4)	C8···H3^v^	3.5002
O1···C14^iv^	3.245 (4)	C7···H2b^i^	3.5859
O1···C15^iv^	3.407 (4)	C6···H7^vi^	3.4036
N1···C9^vi^	3.543 (4)	C5···H8^vi^	3.3410
N1···C10^vi^	3.579 (4)	C4···H1^vii^	3.5635
N1···C11^vi^	3.584 (4)	C4···H7^v^	3.2202
N1···C12^vi^	3.548 (4)	C3···H7^ix^	3.2679
N1···C16^vi^	3.501 (4)	C3···H7^v^	3.1868
N1···C8^vi^	3.504 (4)	C3···H8^ix^	3.5317
N2···O2^iii^	3.317 (4)	C2···H6^v^	3.2008
N2···C9^vi^	3.433 (4)	C2···H7^v^	3.5484
N2···C15^iv^	3.549 (4)	C2···H9^iv^	3.5608
C9···O2^i^	3.513 (4)	C2···H2a	3.4792
C9···O1^v^	3.573 (4)	C1···H6^v^	3.2005
C9···N1^vi^	3.543 (4)	C1···H7^vi^	3.5052
C9···N2^vi^	3.433 (4)	C1···H9^iv^	3.2966
C9···C16^vi^	3.566 (5)	C1···H2a	2.8785
C9···C7^v^	3.531 (4)	H1···Br2^viii^	3.3259
C9···C6^v^	3.494 (5)	H1···C11^ix^	3.2864
C9···C1^v^	3.500 (5)	H1···C13^ix^	3.4564
C10···O1^v^	3.599 (4)	H1···C4^vii^	3.5635
C10···N1^vi^	3.579 (4)	H1···H1^viii^	3.4915
C10···C6^v^	3.435 (4)	H1···H7^ix^	2.4008
C10···C2^v^	3.417 (5)	H1···H7^v^	3.4186
C10···C1^v^	3.216 (4)	H1···H8^ix^	2.6504
C11···N1^vi^	3.584 (4)	H2···Br1^i^	3.0863
C11···C7^vi^	3.438 (5)	H2···O2^i^	2.9874
C11···C6^v^	3.577 (4)	H2···C12^v^	3.3776
C11···C6^vi^	3.455 (4)	H2···C13^vi^	3.4377
C11···C5^v^	3.430 (5)	H2···H8^vi^	3.2389
C11···C4^v^	3.386 (5)	H2···H2a^i^	3.2590
C11···C3^v^	3.503 (5)	H2···H2b^i^	3.0600
C12···N1^vi^	3.548 (4)	H4···C9^v^	3.5576
C12···C7^vi^	3.204 (4)	H4···C9^vi^	3.4525
C12···C6^vi^	3.570 (4)	H4···C10^vi^	3.3478
C12···C5^v^	3.395 (4)	H4···C11^vi^	3.5395
C16···N1^vi^	3.501 (4)	H4···H5^v^	3.3911
C16···C9^vi^	3.566 (5)	H4···H2a	3.1151
C16···C8^vi^	3.504 (4)	H4···H2b^iii^	3.4361
C16···C7^vi^	3.512 (4)	H4···H10^iv^	3.1944
C13···C7^vi^	3.545 (5)	H5···O2^i^	2.5933
C14···O2^iv^	3.461 (5)	H5···O2^v^	3.4645
C14···O1^iv^	3.245 (4)	H5···O1^v^	3.4254
C15···O2^iii^	3.591 (4)	H5···N1^v^	3.5727
C15···O2^iv^	3.441 (4)	H5···N2^vi^	3.1676
C15···O1^iv^	3.407 (4)	H5···C16^vi^	3.4957
C15···N2^iv^	3.549 (4)	H5···C15^vi^	3.5171
C15···C15^iv^	3.553 (5)	H5···H4^v^	3.3911
C8···N1^vi^	3.504 (4)	H5···H2a^i^	3.3155
C8···C16^vi^	3.504 (4)	H5···H2a^v^	3.1946
C8···C8^vi^	3.251 (4)	H5···H2b^i^	3.1505
C8···C7^v^	3.382 (4)	H5···H2b^v^	3.0498
C7···O2^i^	3.235 (4)	H5···H10^i^	2.9431
C7···C9^v^	3.531 (4)	H6···Br1^v^	3.5893
C7···C11^vi^	3.438 (5)	H6···O1^v^	3.4300
C7···C12^vi^	3.204 (4)	H6···O1^vi^	3.5961
C7···C16^vi^	3.512 (4)	H6···C14^i^	3.1729
C7···C13^vi^	3.545 (5)	H6···C15^i^	2.9843
C7···C8^v^	3.382 (4)	H6···C2^v^	3.2008
C6···C9^v^	3.494 (5)	H6···C1^v^	3.2005
C6···C10^v^	3.435 (4)	H6···H9^i^	2.8425
C6···C11^v^	3.577 (4)	H6···H2a^v^	3.2544
C6···C11^vi^	3.455 (4)	H6···H2b^v^	3.0578
C6···C12^vi^	3.570 (4)	H6···H10^i^	2.5189
C5···C11^v^	3.430 (5)	H7···Br2^xi^	3.1930
C5···C12^v^	3.395 (4)	H7···C6^vi^	3.4036
C4···C11^v^	3.386 (5)	H7···C4^v^	3.2202
C3···C11^v^	3.503 (5)	H7···C3^xi^	3.2679
C3···C3^vii^	3.553 (5)	H7···C3^v^	3.1868
C2···C10^v^	3.417 (5)	H7···C2^v^	3.5484
C1···C9^v^	3.500 (5)	H7···C1^vi^	3.5052
C1···C10^v^	3.216 (4)	H7···H1^xi^	2.4008
Br2···H1	2.9246	H7···H1^v^	3.4186
Br2···H2	2.9386	H8···Br2^v^	3.3349
Br1···H1	2.8453	H8···Br1^xi^	3.3429
O1···H4	1.9019	H8···C5^vi^	3.3410
N1···H5	2.6838	H8···C3^xi^	3.5317
N2···H4	2.3054	H8···H1^xi^	2.6504
N2···H9	3.2262	H8···H2^vi^	3.2389
C9···H4	3.1744	H9···Br2^xii^	3.1799
C9···H7	3.2758	H9···Br1^iv^	3.1411
C9···H3	2.6758	H9···O2^iv^	3.4986
C11···H5	3.2439	H9···O1^iv^	2.6114
C11···H8	2.7263	H9···C10^ii^	3.5628
C12···H6	3.2712	H9···C2^iv^	3.5608
C12···H9	3.2986	H9···C1^iv^	3.2966
C16···H4	2.4811	H9···H6^ii^	2.8425
C16···H5	3.2915	H9···H2a^iv^	2.9358
C16···H7	3.2635	H2a···Br1	3.2207
C16···H8	3.3035	H2a···O2^iii^	3.2332
C16···H10	3.1514	H2a···O1	1.8607
C13···H7	2.6451	H2a···C14^iv^	3.1287
C13···H10	3.2352	H2a···C15^iv^	3.2156
C15···H4	3.5810	H2a···C2	3.4792
C15···H8	3.2635	H2a···C1	2.8785
C8···H6	3.2335	H2a···H2^ii^	3.2590
C8···H3	2.6218	H2a···H4	3.1151
C7···H2	2.5738	H2a···H5^ii^	3.3155
C7···H5	2.7450	H2a···H5^v^	3.1946
C6···H4	2.4512	H2a···H6^v^	3.2544
C5···H1	3.2960	H2a···H9^iv^	2.9358
C5···H3	2.5606	H2a···H2b^iii^	3.0958
C3···H2	3.2649	H2a···H10^iv^	3.0931
C1···H1	3.3264	H2a···H3^ii^	3.0138
C1···H2	3.3407	H2b···O2^iii^	2.9125
C1···H4	2.4574	H2b···O1	3.2329
C1···H3	3.3243	H2b···N2^iii^	2.4795
H2···H3	2.3548	H2b···C16^iii^	3.1593
H4···H5	3.4942	H2b···C14^iii^	3.4607
H4···H3	2.6355	H2b···C15^iii^	2.6491
H5···H6	2.2800	H2b···C7^ii^	3.5859
H5···H3	2.1835	H2b···H2^ii^	3.0600
H6···H7	2.3672	H2b···H4^iii^	3.4361
H7···H8	2.5302	H2b···H5^ii^	3.1505
H8···H9	2.3801	H2b···H5^v^	3.0498
H9···H10	2.2819	H2b···H6^v^	3.0578
Br2···H1^viii^	3.3259	H2b···H2a^iii^	3.0958
Br2···H7^ix^	3.1930	H2b···H2b^iii^	3.1439
Br2···H8^v^	3.3349	H2b···H10^iii^	2.7953
Br2···H9^x^	3.1799	H2b···H3^ii^	2.7509
Br1···H2^ii^	3.0863	H10···O2^iv^	3.4704
Br1···H6^v^	3.5893	H10···O1^iv^	2.9448
Br1···H8^ix^	3.3429	H10···N2^iv^	2.7204
Br1···H9^iv^	3.1411	H10···C9^ii^	3.2816
Br1···H2a	3.2207	H10···C10^ii^	3.0491
O2···H2^ii^	2.9874	H10···C15^iv^	2.9333
O2···H5^ii^	2.5933	H10···H4^iv^	3.1944
O2···H5^v^	3.4645	H10···H5^ii^	2.9431
O2···H9^iv^	3.4986	H10···H6^ii^	2.5189
O2···H2a^iii^	3.2332	H10···H2a^iv^	3.0931
O2···H2b^iii^	2.9125	H10···H2b^iii^	2.7953
O2···H10^iv^	3.4704	H10···H10^iv^	2.5278
O2···H3^ii^	2.3043	H3···O2^i^	2.3043
O1···H5^v^	3.4254	H3···C12^vi^	3.2196
O1···H6^v^	3.4300	H3···C16^vi^	3.3870
O1···H6^vi^	3.5961	H3···C13^vi^	3.2974
O1···H9^iv^	2.6114	H3···C14^vi^	3.4954
O1···H2a	1.8607	H3···C8^v^	3.5002
O1···H2b	3.2329	H3···H2a^i^	3.0138
O1···H10^iv^	2.9448	H3···H2b^i^	2.7509
			
C8—N1—C7	126.9 (3)	Br1—C2—C1	118.04 (19)
C16—N2—C15	117.5 (3)	C3—C2—C1	122.9 (3)
C10—C9—C8	119.6 (3)	O1—C1—C6	122.4 (3)
C9—C10—C11	120.9 (3)	O1—C1—C2	123.1 (3)
C10—C11—C12	120.9 (3)	C6—C1—C2	114.5 (3)
C11—C12—C16	119.0 (3)	H2a—O2—H2b	104.997
C11—C12—C13	124.0 (3)	C8—N1—H4	118.703
C16—C12—C13	117.0 (3)	C7—N1—H4	114.421
N2—C16—C12	123.1 (3)	C10—C9—H5	118.165
N2—C16—C8	117.8 (3)	C8—C9—H5	122.173
C12—C16—C8	119.1 (3)	C9—C10—H6	117.050
C12—C13—C14	119.8 (3)	C11—C10—H6	122.057
C13—C14—C15	118.6 (3)	C10—C11—H7	122.229
N2—C15—C14	123.9 (3)	C12—C11—H7	116.883
N1—C8—C9	123.8 (3)	C12—C13—H8	121.326
N1—C8—C16	115.6 (3)	C14—C13—H8	118.840
C9—C8—C16	120.5 (3)	C13—C14—H9	125.339
N1—C7—C6	122.7 (3)	C15—C14—H9	116.025
C7—C6—C5	117.7 (3)	N2—C15—H10	118.045
C7—C6—C1	120.1 (3)	C14—C15—H10	118.053
C5—C6—C1	122.0 (3)	N1—C7—H3	118.643
C6—C5—C4	119.2 (3)	C6—C7—H3	118.650
Br2—C4—C5	120.9 (2)	C6—C5—H2	119.587
Br2—C4—C3	117.73 (19)	C4—C5—H2	120.964
C5—C4—C3	121.2 (3)	C4—C3—H1	121.379
C4—C3—C2	120.2 (3)	C2—C3—H1	118.393
Br1—C2—C3	119.0 (2)		
			
C8—N1—C7—C6	178.3 (3)	C12—C16—C8—C9	−0.6 (4)
C7—N1—C8—C9	−3.4 (5)	C12—C13—C14—C15	0.6 (5)
C7—N1—C8—C16	176.7 (3)	C13—C14—C15—N2	1.0 (5)
C16—N2—C15—C14	−1.3 (5)	N1—C7—C6—C5	−178.0 (3)
C15—N2—C16—C12	−0.1 (4)	N1—C7—C6—C1	−2.1 (5)
C15—N2—C16—C8	−178.2 (3)	C7—C6—C5—C4	176.6 (3)
C10—C9—C8—N1	−179.5 (3)	C7—C6—C1—O1	2.5 (5)
C10—C9—C8—C16	0.4 (4)	C7—C6—C1—C2	−176.5 (3)
C8—C9—C10—C11	0.0 (5)	C5—C6—C1—O1	178.2 (3)
C9—C10—C11—C12	−0.3 (5)	C5—C6—C1—C2	−0.7 (4)
C10—C11—C12—C16	0.1 (5)	C1—C6—C5—C4	0.8 (5)
C10—C11—C12—C13	−179.3 (3)	C6—C5—C4—Br2	−175.0 (3)
C11—C12—C16—N2	−177.8 (3)	C6—C5—C4—C3	0.1 (5)
C11—C12—C16—C8	0.3 (4)	Br2—C4—C3—C2	174.29 (19)
C11—C12—C13—C14	177.5 (3)	C5—C4—C3—C2	−1.0 (5)
C16—C12—C13—C14	−1.8 (4)	C4—C3—C2—Br1	−175.4 (3)
C13—C12—C16—N2	1.6 (4)	C4—C3—C2—C1	1.0 (5)
C13—C12—C16—C8	179.7 (3)	Br1—C2—C1—O1	−2.7 (4)
N2—C16—C8—N1	−2.5 (4)	Br1—C2—C1—C6	176.26 (16)
N2—C16—C8—C9	177.6 (3)	C3—C2—C1—O1	−179.2 (3)
C12—C16—C8—N1	179.3 (3)	C3—C2—C1—C6	−0.2 (4)

Symmetry codes: (i) *x*, *y*−1, *z*; (ii) *x*, *y*+1, *z*; (iii) −*x*, −*y*+1, −*z*+2; (iv) −*x*+1, −*y*+1, −*z*+2; (v) −*x*, −*y*, −*z*+2; (vi) −*x*+1, −*y*, −*z*+2; (vii) −*x*, −*y*, −*z*+1; (viii) −*x*−1, −*y*, −*z*+1; (ix) *x*−1, *y*, *z*−1; (x) *x*−1, *y*−1, *z*−1; (xi) *x*+1, *y*, *z*+1; (xii) *x*+1, *y*+1, *z*+1.

### Hydrogen-bond geometry (Å, °)

**Table:**

*D*—H···*A*	*D*—H	H···*A*	*D*···*A*	*D*—H···*A*
O2—H2a···O1	0.95	1.86	2.802 (4)	171.
N1—H4···O1	0.83	1.90	2.592 (4)	140.
N1—H4···N2	0.83	2.31	2.668 (3)	107.
C7—H3···O2^i^	0.95	2.31	3.234 (4)	166.

Symmetry codes: (i) *x*, *y*−1, *z*.
